# Wm. R. Warner & Co.

**Published:** 1889-07

**Authors:** 


WM. R. WARNER & Co.—We had the pleasure, recently, of a call from Dr. H. M. Pinkard, a genial and intelligent gentleman from the House of Wm. R. Warner & Co., of Philadelphia, who kindly presented us with rare samples of some of their excellent preparations. Among the samples presented was the *Podophyllin Parvules*, so popular as a remedy in constipation, etc.; and his Antiseptic Pastiles, being Carl Seiler's antiseptic formula put into tablet form for the convenience of practitioners.

The neatness and reliability of Warner's preparations have secured for their House the confidence of the profession everywhere.

